# Dietary carbohydrate quality index and incidence of obesity-related cancers in the “Seguimiento Universidad De Navarra” (SUN) prospective cohort

**DOI:** 10.1007/s00394-024-03438-3

**Published:** 2024-05-30

**Authors:** M. Olmedo, S. Santiago, A. Romanos-Nanclares, J. M. Aramendia-Beitia, R. Sanchez-Bayona, M. Bes-Rastrollo, M. A. Martinez-Gonzalez, E. Toledo

**Affiliations:** 1 Department of Preventive Medicine and Public Health, University of Navarra, C/ Irunlarrea, 1, Pamplona, Pamplona, 31008 Spain; 2 Department of Medical Oncology, Cancer Center Clínica Universidad de Navarra, Pamplona, Spain; 3 Navarra Institute for Health Research (IdiSNA), Pamplona, Spain; 4 CIBERobn, Instituto de Salud Carlos III, Madrid, Spain; 5 Department of Nutrition and Food Sciences and Physiology, University of Navarra, Pamplona, Spain; 6 Channing Division of Network Medicine, Department of Medicine, Brigham and Women’s Hospital, Harvard Medical School, Boston, MA USA; 7 Medical Oncology Department, Hospital Universitario 12 de Octubre, Madrid, Spain; 8 Department of Nutrition, Harvard T.H. Chan School of Public Health, Boston, USA

**Keywords:** Obesity-related cancer, Fiber, Glycemic index, Whole grain, Liquid carbohydrates

## Abstract

**Purpose:**

The quality, rather than the quantity, of carbohydrate intake may play a major role in the etiology of obesity-related cancers (ORCs). We assessed the association between a previously defined carbohydrate quality index (CQI) and the risk of developing ORCs in the “Seguimiento Universidad de Navarra” (SUN) cohort.

**Methods:**

A total of 18,446 Spanish university graduates [mean age 38 years (SD 12 years), 61% women, mean BMI 23.5 kg/m^2^ (SD 3.5 kg/m^2^)], with no personal history of cancer, were followed-up. Baseline CQI was assessed summing quintiles of four previously defined criteria: high dietary fiber intake, low glycemic index (GI), high whole-grain: total-grain carbohydrates ratio and high solid carbohydrates: total carbohydrates ratio. Participants were classified into tertiles of their total CQI. Incident ORCs were confirmed by an oncologist using medical records and by querying the National Death Index blindly to dietary exposures.

**Results:**

During a median follow-up of 13.7 years, 269 incident cases of ORC were confirmed. A higher CQI was inversely associated with ORC incidence [multivariable-adjusted hazard ratio (HR) for the upper (T3) versus the lowest tertile (T1) of 0.68 (95% CI: 0.47–0.96), p for trend = 0.047]. Particularly, higher dietary fiber intake was inversely associated with ORC, HR_T3 vs. T1_=0.57 (95% CI 0.37–0.88 p for trend = 0.013).

**Conclusion:**

In this prospective Mediterranean cohort, an inverse association between a better global quality of carbohydrate intake and the risk of ORCs was found. Strategies for cancer prevention should promote a higher quality of carbohydrate intake.

## Introduction

Obesity is an important public health issue, with its prevalence alarmingly rising in recent years to reach epidemic proportions. Worldwide, over a billion people suffer from obesity, including 650 million adults, 340 million adolescents, and 39 million children. This figure has been steadily increasing. According to estimates from the World Health Organization, by 2025, around 167 million individuals, including both adults and children, will face health issues due to being overweight or obese [1]. Obesity is defined as excessive fat accumulation that might impair health and is diagnosed at a body-mass index (BMI) ≥ 30 kg/m^2^ [2]. Overweight and obesity are risk factors for numerous chronic diseases, such as cardiovascular disease and cancer, the first and second leading causes of death worldwide. Cancer is the leading cause of death in developed countries, and its disease burden is expected to increase as the population ages. Actually, obesity is quickly becoming the primary preventable cause of cancer, surpassing tobacco. Worldwide, it has been estimated that obesity contributes to about 4–8% of all cancers, ranging from less than 1% in low-income countries to 7–8% in high-income countries [3]. In Europe, obesity is estimated to be directly responsible for at least 200,000 new cancer cases per year [4]. There is strong evidence that maintaining a healthy weight reduces the risk of certain types of tumors. Specifically, a report from the International Agency for Research on Cancer (IARC) has identified 13 types of cancer associated with overweight and obesity: meningioma, esophageal adenocarcinoma, multiple myeloma, and kidney, endometrial, ovarian, thyroid, postmenopausal breast, liver, gallbladder, stomach, pancreatic and colorectal cancer [5].

Although several mechanisms have been postulated to explain the possible association between obesity and cancer, the role of obesity in cancer etiopathogenesis has not been fully elucidated [6, 7]. Understanding how nutrition affects cancers linked to obesity will be crucial. It can help identify individuals at higher risk and develop personalized strategies for cancer prevention. The main mechanisms that seem to be implicated are insulin resistance, adipokine aberration, subclinical chronic low-grade inflammation and oxidative stress [8]. Specifically, the relationship between insulin resistance and the development of obesity has been extensively studied [9].

Rather than the total amount of carbohydrates in the diet, the quality of carbohydrates seems to play a more important role in the etiology of chronic diseases [10]. Quantifying carbohydrate quality is complex. Therefore, Zazpe et al. [11] proposed using a multidimensional carbohydrate quality index (CQI) that integrates four characteristics: the glycemic index, the ratio of whole grains to total grain carbohydrates, the ratio of solid carbohydrates to total carbohydrates and total dietary fiber intake. This index has previously been inversely associated with changes in weight in the PREDIMED-Plus study [12] and in the SUN Project [13] and with breast cancer incidence in the SUN Project [14], but its possible association with obesity-related cancers has not yet been evaluated.

Therefore, our aim was to explore how the overall quality of carbohydrate intake relates to the risk of developing obesity-related cancers in a large prospective Mediterranean cohort.

## Materials and methods

### Study population

The ‘Seguimiento Universidad de Navarra’ (SUN) Project is an ongoing, multipurpose, cohort study composed of university graduates in Spain, that aims to determine the association between diet and the occurrence of major chronic diseases [15]. The recruitment began in 1999 and is ongoing. When participants were recruited, they completed a baseline questionnaire, which collected information about lifestyle, sociodemographic, anthropometric and medical variables. They were contacted biennially through follow-up questionnaires to update their medical conditions [16].

The present study was conducted following the guidelines of the Declaration of Helsinki and the procedures were approved by the Institutional Review Board of the University of Navarra (approval code 010830). Voluntary fulfillment of the baseline questionnaire was considered as informed consent. This cohort is registered at clinicaltrials.gov as NCT02669602.

**Fig. 1:**
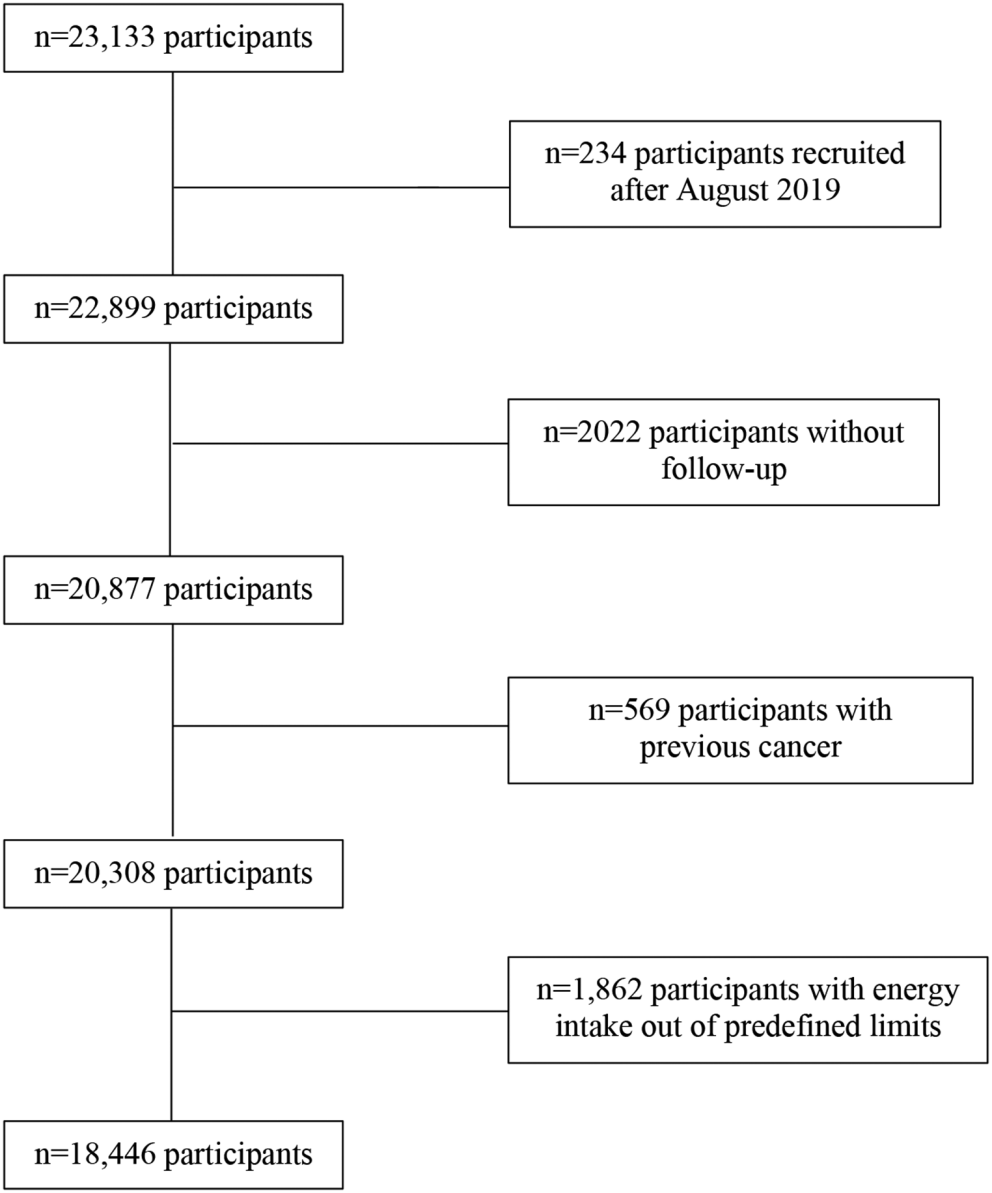
Flow-chart of participants In the SUN project. 1999–2022

Through May 2022, 23,133 participants had been recruited and had completed the baseline questionnaire of the SUN Project. For this analysis, we used the following exclusion criteria: 234 participants who answered the baseline questionnaire after August 1st, 2019 were excluded to ensure a minimum 2-year follow up period; 2,022 participants were excluded due to lack of follow-up information; we further excluded 569 participants who reported a previous cancer diagnosis at the time of recruitment. Lastly, we excluded 1,862 participants with a daily energy intake out of the predefined limits (below 500 kcal/day or above 3500 kcal/day for women, and below 800 kcal/day or above 4000 kcal/day for men) [17]. Thus, the final sample was 18,446 participants (Fig. 1).

### Dietary assessment

At baseline, participants completed a validated 136-item semi-quantitative food frequency questionnaire (FFQ) [18]. Previously published reproducibility and validity of this FFQ showed reasonably good validity for assessing carbohydrate intake [19]. There were 9 options for the average frequency of consumption (from “never or seldom” to “> 6 times/day”) and trained dietitians updated the nutrient data bank according to the information from Spanish food composition tables [20, 21].

The CQI has been previously used in other studies assessing nutritional adequacy, weight gain, incident breast cancer and cardiovascular disease [11, 13, 14, 22]. We calculated the CQI at baseline using the method proposed by Zazpe et al. [11]. Liquid carbohydrate intake was estimated adding up sugar-sweetened beverages and fruit juices consumption, while solid carbohydrate intake corresponded to the rest of foods with any carbohydrate content. Refined grains were determined according to 13 items linked to the consumption of white rice, refined bread, refined pasta and different bakery products made with different refined grains; and whole grain consumption was ascertained with the item “whole bread consumption”, as used in other studies of our group [14].

The CQI was defined by adding up the –roughly– quintiles for the following four criteria: dietary fiber intake (g/day, positively weighted), glycemic index (negatively weighted), ratio whole grains: total grains carbohydrate (positively weighted), and lastly, solid carbohydrates: total carbohydrates ratio (solid + liquid carbohydrates) (positively weighted) [11]. All criteria had the same weight, and the total score theoretically ranged from 4 to 20, with higher CQI scores denoting better carbohydrate quality. The CQI was then categorized into roughly tertiles (4–10 / 11–13 / 14–20 points).

### Ascertainment of obesity-related cancer cases

The primary endpoint for this study was the diagnosis of incident tumors included in the 13 types of cancer associated with overweight and obesity according to previous evidence [5]: esophageal adenocarcinoma (C15 M-8140/3), postmenopausal breast carcinoma (C50 M-8000/3), colon and rectum (C18 M-8000/3 and C19 M8000/3), uterus (C54.1 M-8000/3), stomach (C16 M8000/3), kidney (C64.9 M8000/3), liver (C22 M8000/3), biliary tract cancer (C23 M8000/3 and C24 M8000/3), ovary (C56 M8000/3), pancreas (C25 M8140/3), thyroid (C73.9 M8260/3), meningioma (C70 M9530/0), and multiple myeloma (C42.1 M9732/3).

Participants who reported any of these diagnoses were asked to provide a copy of their medical records. Afterward, an independent oncologist, who was unaware of the exposure, confirmed the cases by reviewing these records. Participants who did not provide a medical record were asked for consent to be contacted by telephone by an oncologist to confirm if they had malignancy. Deaths due to any cancer identified by reviewing the National Death Index were also included as confirmed cases.

### Assessment of other variables

The baseline questionnaire included information on sociodemographic, lifestyle and medical variables. BMI was calculated as weight (in kilograms)/square of height (in meters), using self-reported information, whose accuracy to estimate the BMI has been previously validated in this cohort [23]. Information on physical activity during leisure-time was collected at baseline with a specific questionnaire previously validated in Spain [24]. To assess leisure-time physical activity, an activity metabolic equivalent (MET) index was computed by designating a multiple of resting metabolic rate (MET score) to each activity and calculating overall MET-hours per week. The adherence to the Mediterranean dietary pattern was evaluated using the Mediterranean Diet Score proposed by Trichopoulou et al. [25], excluding the alcohol intake component, due to its association with several obesity-related cancers.

### Statistical analyses

We described the baseline characteristics of participants according to CQI tertiles with means (SD) for quantitative traits and percentages for qualitative ones.

To assess the relationship between CQI and the risk of an obesity-related cancer, we used Cox regression models, with the lowest tertile as the reference category. The models included age as an underlying time variable and were additionally stratified by recruitment period and age (decades) at recruitment. Time at entry was defined as the date of completion of the baseline questionnaire.

Participants were followed-up until they developed an obesity-related cancer –as for the cases– or until the date of death due to a cause other than an obesity-related cancer, the date of completion of the last returned questionnaire or the last contact, whichever happened later, –as for the non-cases–.

We also used the CQI as a continuous variable and assessed the hazard ratios for the risk of an obesity-related cancer associated with a two-point increase in the CQI. In addition, we repeated Cox regression models using individual components of CQI: dietary fiber intake, glycemic index (GI), ratio of whole-grain: total-grain carbohydrates and solid carbohydrates: (solid carbohydrates + liquid carbohydrates) ratio.

We fitted two multivariable models for all exposures, adjusting for the main obesity-related cancer risk factors and other potential selected confounders, based on existing evidence and previous results of our cohort studies on cancer [14, 26–30]. Model 1 included sex, BMI (continuous). height (continuous), years of university studies (continuous), family history of breast or colorectal cancer (yes/no), smoking status (never smoker, former smoker, current smoker), lifetime tobacco consumption (pack-years, continuous), physical activity (METs-h/week, tertiles), and alcohol intake (g/d, continuous). Model 2 was additionally adjusted for total energy intake (continuous), adherence to the Mediterranean diet (continuous), following a special diet (yes/no), previous colonoscopy (yes/no), previous mammography (yes/no), and diabetes at baseline (yes/no).

Tests of linear trend across successive tertiles of CQI and individual components of CQI were conducted assigning the median value to each category and treating the resulting variables as continuous. We also estimated the p-value obtained for the independent variable (CQI, range 4 to 20) introduced in the model as a continuous variable.

We assessed potential non-linear associations with restricted cubic splines.

The proportionality of the hazards was checked testing for a non-zero slope in a generalized linear regression of the scaled Schoenfeld residuals on functions of time.

We also assessed the interaction between the carbohydrate quality and quantity with a likelihood ratio test with 2 degrees of freedom [tertiles of CQI and 2 categories of energy intake provided by carbohydrates (≤ 50%, > 50% of total energy intake)].

As sensitivity analyses, we recalculated the CQI without the fiber component and we repeated our analyses excluding the three most frequent cancers (colorectal, breast and pancreatic) and diabetic participants. We also repeated our analyses for the most frequent cancers individually. Additionally, we stratified the analyses by sex (male or female), age (< 50 years, ≥50 and < 70 years or ≥70 years), BMI (< or ≥ 25 kg/m^2^), or follow-up (followed until death or censored).

Confidence intervals were estimated with 95% confidence and a two-sided p value < 0.05 was deemed as statistically significant.

## Results

We included 18,446 participants for the analyses who accrued 238,966 person-years of follow-up. Table 1 shows baseline characteristics of participants according to tertiles of the CQI. Overall, 61% of the participants were female and mean age was 38 years (SD 12 years). The mean BMI was 23.5 kg/m^2^ (SD 3.5 kg/m^2^). Approximately 30% were former smokers and 21% current smokers.

**Table 1:** Baseline characteristics of participants according to tertiles of the carbohydrate quality index: the ‘Seguimiento Universidad de Navarra’ (SUN) Project: 1999–2019

Variable	Tertile 1	Tertile 2	Tertile 3
n	7932	5759	4755
CQI range (points)	4–10	11–13	14–20
Age (years)	37 (12)	38 (12)	40 (12)
Sex (%)			
Male	46.1﻿	39.1	31.1
Female	53.9﻿	60.9	68.9
Body mass index (kg/m^2^)	23.6 (3.6)	23.6 (3.5)	23.4 (3.5)
Alcohol intake (g/d)	7.4 (11.6)	6.5 (9.7)	5.7 (8.2)
Height (cm)	169 (9)	168 (9)	167 (8)
Smoking (%)			
Never smoker	﻿46.8	50.5	50.1
Current smoker	﻿25.8	20.3	17.3
Former smoker	27.4	29.3	32.5
Diabetes (%)	1.5	1.75	2.59
Tobacco consumption (packs-year)	6.4 (10.3)	5.9 (9.6)	5.9 (9.5)
Family history of breast cancer (%)	26.3	27.0	27.1
Family history of colon cancer (%)	14.4	16.2	15.0
TV watching (hours/day)	1.6 (1.2)	1.5 (1.2)	1.5 (1.2)
Years of university studies	5.0 (1.5)	5.1 (1.5)	5.1 (1.5)
Adherence to the Mediterranean diet [25]	3.3 (1.6)	4.2 (1.6)	5.1 (1.5)
Special diet (%)	5.9	8.1	12.6
Total energy intake (kcal/day)	2229 (607)	2401 (625)	2463 (591)
Physical activity (METs-h/week)	20 (21)	22 (23)	26 (26)
Fiber intake (g/day)	16.6 (5.5)	23.6 (8.2)	31.2 (10.6)
Glycemic index	52.9 (4.8)	51.6 (4.6)	50.8 (4.3)
Solid carbohydrate intake (g/day)	201 (72)	232 (81)	250 (78)
Liquid CH intake (g/day)	36 (22)	31 (20)	27 (19)
CH from whole grains intake (g/day)	0 (3)	5 (11)	19 (23)
CH from refined grains intake (g/day)	91 (51)	92 (56)	77 (47)

Values are expressed as mean (SD) unless otherwise stated

CQI: carbohydrate quality index; MET: metabolic equivalent task; CH: carbohydrate

Participants with a higher CQI were more likely to be female and older. They also showed higher adherence to the Mediterranean diet, higher energy intake, and higher levels of physical activity. Alcohol intake was higher among participants with lower CQI score. Other characteristics such as BMI and family history of breast or colorectal cancer were similar across groups.

During a median follow-up of 13.7 years, we identified 269 new-onset obesity-related cancers cases (71 postmenopausal breast, 56 colon, 24 rectum, 11 uterus, 11 ovarian, 33 pancreatic, 7 esophageal, 14 stomach, 12 biliary tract cancers, 5 hepatocellular carcinomas, 4 multiple myelomas, 1 meningioma, 16 renal cell carcinomas, and 26 thyroid carcinomas) with 22 participants developing two obesity-related cancers. Those participants in the highest tertile of CQI had a significantly lower risk of developing cancer during the follow-up period compared to their counterparts in the lowest tertile (HR_T3 vs. T1_ = 0.68, 95% CI 0.47–0.96) in the fully adjusted model (Table 2). The p for trend in the fully adjusted model was 0.047. When the fiber component was not included in the CQI, the association was slightly attenuated (HR_T3 vs. T1_ 0.78 (95%CI 0.58–1.05)). The exposure adequately met the assumption of proportionality of the hazards.

**Table 2:** Hazard ratios and 95% confidence intervals for confirmed obesity-related cancers (*n* = 269) by tertiles of carbohydrate quality indexa in the SUN Project (1999–2019)

Confirmed cases	Tertiles of CQI				For each additional 2 points in the score
	Tertile 1	Tertile 2	Tertile 3	*p for trend*	
N	7932	5759	4755		
CQI range	4–10	11–13	14–20		
Incident cases	114	98	57		
Person-years	105,494	74,611	58,862		
Sex and age-adjusted	1 (ref.)	1.00 (0.76–1.32)	0.63 (0.46–0.87)	0.009	0.93 (0.86-1.00)
Model 1	1 (ref.)	1.07 (0.82–1.41)	0.71 (0.51–0.99)	0.071	0.96 (0.88–1.04)
Model 2	1 (ref.)	1.06 (0.80–1.41)	0.68 (0.47–0.96)	0.047	0.95 (0.87–1.04)

Model 1 additionally adjusted for sex, height (continuous), years at university (continuous), family history of breast or colorectal cancer (yes/no), smoking status (never smoker, former smoker, current smoker), lifetime tobacco consumption (continuous), physical activity (METs-h/ week, tertiles), alcohol intake (continuous), BMI (continuous). **Model 2** additionally adjusted for energy intake (continuous), adherence to the Mediterranean diet (kcal/day, continuous), special diet (yes/no), colonoscopy (yes/no), mammography (yes/no), diabetes at baseline (yes/no). ^a^ Participants were categorized according to the following items: dietary fiber intake (g/day), Glycemic Index (GI), ratio whole grains: total grains carbohydrate (positively weighted), and ratio solid carbohydrates: total carbohydrates (solid + liquid carbohydrate). For each of them, participants were categorized into quintiles and received their values. The four criteria had the same weight, and the score range was from 4 to 20, the higher CQI meaning better carbohydrate quality

The restricted cubic splines analyses suggested no deviation from linearity (p for non-linearity = 0.24).

There was no statistically significant interaction between the tertiles of CQI and the carbohydrate quantity.

**Fig. 2:**
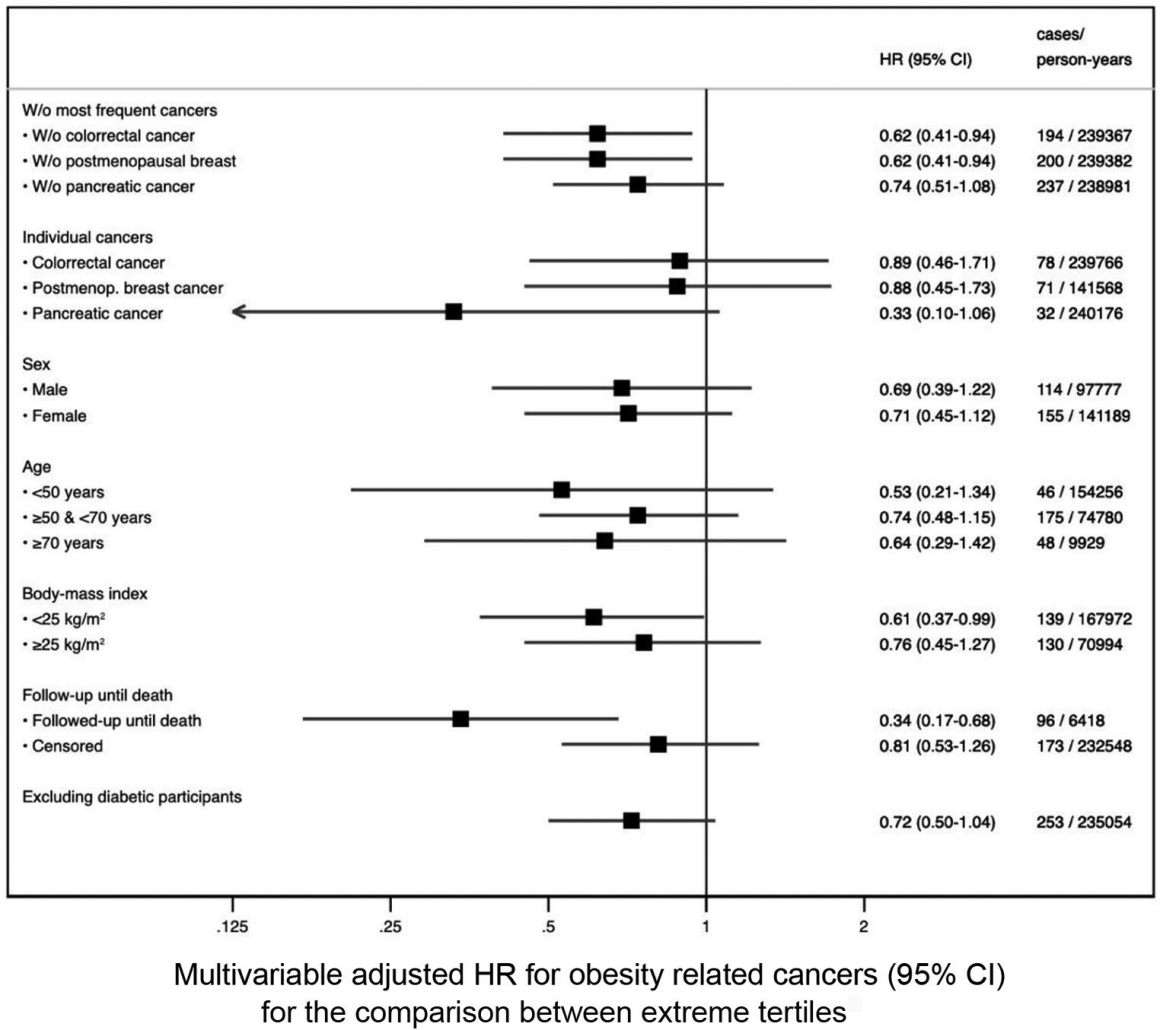
Results from sensitivity analyses for participants in the third tertile of adherence to the carbohydrate quality index compared to participants in the first tertile. Note. w/o: without

Stratified analyses by sex (‘male’ and ‘female’), age (‘<50 years’, ‘50–70 years’ and ‘>70 years’) and BMI (‘normal weight’ and ‘overweight and obesity’) did not reveal any substantial differences across subgroups and no statistically significant interactions were found. We also performed sensitivity analyses excluding the most frequent obesity-related cancers in the sample (colorectal, breast and pancreatic cancers), and results barely changed (Fig. 2).

Table 3 shows the incidence of obesity-related cancers during follow-up according to the comparisons between the highest and the lowest tertile of each individual component of CQI. A higher dietary fiber intake (tertile 3) was associated with a 43% relative risk reduction of obesity-related cancers (HR_T3 vs. T1_= 0.57, 95% CI 0.37–0.88).

**Table 3:** Hazard ratios and 95% confidence intervals for confirmed obesity-related cancers by tertiles of individual components of carbohydrate quality index in the SUN Project (1999–2019)

	Tertiles of glycemic index				
	Tertile 1	Tertile 2		Tertile 3	*p for trend*
N	6149	6149		6148	
Incident cases	100	93		76	
Person-years	77,393	79,283		82,291	
Multivariable adjusted model	1 (ref.)	1.25 (0.93–1.67)		0.99 (0.72–1.37)	0.969
	Tertiles of dietary fiber intake (g/d)				
	Tertile 1	Tertile 2		Tertile 3	*p for trend*
N	6149	6149		6148	
Incident cases	95	87		87	
Person-years	80,012	80,814		78,140	
Multivariable adjusted model	1 (ref.)	0.74 (0.53–1.04)		0.57 (0.37–0.88)	0.011
Tertiles of solid carbohydrates: (solid carbohydrates + liquid carbohydrates) ratio					
	Tertile 1	Tertile 2		Tertile 3	*p for trend*
N	6149	6149		6148	
Incident cases	88	80		101	
Person-years	79,388	79,889		79,689	
Multivariable adjusted model	1 (ref.)	0.97 (0.71–1.32)		1.07 (0.79–1.44)	0.653
	Intake of whole grain: total grain carbohydrates ratio				
	No whole grain intake		Some whole grain intake		*p for trend*
N	11589		6857		
Incident cases	183		86		
Person-years	153,462		85,505		
Multivariable adjusted model	1 (ref.)		0.86 (0.66–1.12)		0.260

Multivariable adjusted model adjusted for sex, age, height (continuous), years at university (continuous), family history of breast or colorectal cancer (yes/no), smoking status (never smoker, former smoker, current smoker), lifetime tobacco consumption (continuous), physical activity (METs-h/ week, tertiles), alcohol intake (continuous), BMI (continuous), energy intake (continuous), adherence to the Mediterranean diet (kcal/day, continuous), special diet (yes/no), colonoscopy (yes/no), mammography (yes/no), diabetes at baseline (yes/no)

## Discussion

In this prospective cohort study, we found that participants with a higher CQI had a lower risk of obesity-related cancers. The CQI is a multidimensional score based on the dietary fiber, the GI, the form of carbohydrate intake (solid or liquid) and the degree of processing of carbohydrates-rich foods (whole or refined grains), using a previously reported definition [11]. When analyzing these factors individually, we found that a higher intake of dietary fiber was also associated with a lower risk of obesity-related cancers.

The quantity, quality, and dietary sources of carbohydrates, as well as their proportion in total energy intake, have become a growing concern in cancer prevention. Traditionally, GI and GL have been commonly used as indicators of carbohydrate quality. However, recent discussions suggest that fiber intake and the consumption of whole grains could also serve as appropriate markers of carbohydrate quality [31, 32].

Different studies have evaluated the association between the quality of carbohydrates in the diet, measured through various indicators, and the risk of developing cancer. A meta-analysis of prospective studies and clinical trials examined the relationship between different indicators of carbohydrate quality (fiber intake, whole grain consumption, GI, or GL; all included in the index used in our study) and the incidence of various chronic diseases. The meta-analysis concluded that higher consumption of fiber or whole grains was associated with a reduction in the risk of incidence and mortality from different non-communicable diseases. Specifically, prospective studies revealed a decrease in the incidence of colorectal, breast, and esophageal cancer associated with higher fiber intake. Results regarding whole grain consumption were consistent [33]. According to the evidence, dietary glycemic index or glycemic load might be less useful as overall measures of carbohydrate quality, which is consistent with our study, as we found that considering individual components of the index, a higher dietary fiber intake was inversely associated with the incidence of obesity-related cancers.

Other cohort studies have examined the relationship between different markers of carbohydrate quality and the risk of cancer. In the NutriNet Santé cohort, the association between glycemic index and glycemic load and the risk of developing cancer was investigated. The authors concluded that a higher dietary glycemic load was associated with a higher overall cancer risk and specifically with postmenopausal breast cancer [34]. Another cohort study evaluated multiple types of dietary fiber intake on mortality outcomes and found a significant inverse association between total dietary fiber intake and cancer mortality [35].

On the other hand, several studies have assessed the association between different indicators of carbohydrate quality and the risk of developing individual cancers. Romanos-Nanclares et al. evaluated the quality of carbohydrates in the diet using the CQI and the risk of developing breast cancer in the SUN cohort, concluding that a higher index was inversely associated with the incidence of breast cancer [14]. This index has also been previously associated with a lower risk of cardiovascular disease [22], and overweight or obesity [13]. A higher CQI has also been associated with a significantly lower risk of colorectal cancer [36]. However, to date, the link between quality of dietary carbohydrates and the risk of developing obesity-related cancer has not been comprehensively studied. When excluding the most frequent cancers in the sensitivity analysis (colorectal, breast and pancreatic cancers), results barely changed; therefore it seems that the inverse association between the CQI and the risk of obesity-related cancers may not be exclusively driven by the most incident tumors, but by the tumors associated with overweight and obesity as a whole.

Obesity activates numerous oncogenic signaling pathways that promote tumor cell survival, proliferation, and metabolism. The molecular mechanisms that explain the obesity and cancer connection include abnormalities in the IGF1 axis and hyperinsulinemia and insulin resistance, altered steroid metabolism which leads to higher concentrations of estrogen levels from peripheral aromatization in adipose tissue, subclinical chronic low-grade inflammation, oxidative stress, alterations in adipocytokine pathophysiology, and disruptions in the microenvironment, such as vascular changes or epithelial-mesenchymal transition [8]. The role of the quantity and quality of carbohydrates in developing overweight and obesity has received major attention. It is therefore intriguing to explore whether they might be linked to the risk of obesity-related cancers and which mechanisms could be involved in this connection. The glycemic index has been associated with an increased risk of postmenopausal breast cancer; the insulin receptor has been shown to be elevated in breast cancer, which may explain this tumor’s sensitivity to hyperinsulinemia [37]. Furthermore, other tumors, including colon, ovary and thyroid cancers, express the insulin receptor in high levels [8].

In our study, we observed an inverse association between a higher consumption of dietary fiber and the risk of developing obesity-related cancers. Previous research has shown that consuming enough dietary fiber may reduce the risk of colorectal and breast cancer [33] and this may be, at least partially, explained by several mechanisms including blood sugar stabilization, insulin response improvement and lower concentration of inflammatory biomarkers [35]. A recent umbrella review showed that dietary fiber provides a protective effect against various types of malignancies, including gastrointestinal cancers (colorectal, gastric and esophageal cancers) and female-specific malignancies (breast, endometrial and ovarian cancers), as well as pancreatic, prostate, and renal cell cancer [38].

Our study has several limitations that should be acknowledged. First, it is important to note that the majority of participants (69.8%) in our cohort are young and physically active, with a mean BMI < 25 kg/m^2^. These characteristics may have contributed to the relatively low incidence of cancer observed in our study, potentially limiting the statistical power. Second, it is crucial to consider that the concept of obesity-related cancer encompasses tumors with diverse pathobiology, carcinogenesis, and cellular pathways. The biological diversity within this group of cancers may also limit our ability to draw definitive conclusions [39]. Third, information on the exposure relied on a self-reported FFQ which could have led to a non-differential misclassification and measurement errors and may probably underestimate true associations. Fourth, our definition of the CQI may not capture all elements of the carbohydrate intake in the context of overall dietary pattern. Fifth, the CQI is defined by adding up roughly quintiles due to ties, especially in the whole grains item. Sixth, the outcome assessment relied on self-reported data, which may result in an underreporting of incident cancer cases and, consequently, lower sensitivity. Nonetheless, it is worth noting that the confirmed cases were independently verified by an oncologist, ensuring high specificity. Seventh, due to the unavailability of socioeconomic status data for the participants, we included years of university studies as a proxy in our multivariable analysis. In addition, our study sample exclusively comprised university graduates and this homogeneity in educational and socioeconomic status may help reduce the potential confounding effect. Lastly, despite the adjustment for a wide range of potential confounders, some residual confounding cannot be completely ruled out given the observational nature of our study design.

On the other hand, the present study has several strengths. We used data obtained from a well-established Mediterranean cohort, specifically the SUN study cohort, which provided a relatively large sample size and achieved a high response rate. Additionally, we were able to adjust for numerous potential confounders in our analysis. Moreover, the measurement of dietary intake was conducted using a previously validated FFQ [19] in a highly educated cohort, ensuring high-quality self-reported data.

In conclusion, in this prospective Mediterranean cohort study, the multidimensional definition of CQI showed a significant inverse association with the incidence of obesity-related cancers during the follow-up period. These findings highlight the importance of prioritizing carbohydrate quality in public health strategies aimed at cancer prevention, such as increasing dietary fiber, whole grain consumption, preferring solid carbohydrates and choosing low GI foods.
